# Neoteny and Evolutionary Strategy: Reconsidering Developmental Arrest in the Axolotl and the Olm

**DOI:** 10.1007/s10441-026-09535-6

**Published:** 2026-07-23

**Authors:** Martine Mussies

**Affiliations:** Maastricht University, Minderbroedersberg 4-6, 6211 LH Maastricht, The Netherlands

**Keywords:** Neoteny, Heterochrony, Amphibian development, Endocrine regulation, Axolotl (*Ambystoma mexicanum*), Olm (*Proteus anguinus*), Regressive evolution, Adaptation, Evolutionary developmental biology (evo-devo), Normativity in biology, Ecological niche, Developmental plasticity

## Abstract

This article examines neoteny and regressive evolution in two emblematic amphibian species: *Ambystoma mexicanum* (axolotl) and *Proteus anguinus* (olm). Rather than framing larval persistence and trait loss as evolutionary shortcomings, the paper proposes an alternative interpretation in which developmental arrest functions as a viable population-level outcome of selection under specific ecological constraints, rather than as a developmental shortcoming. Drawing on evo–devo literature, endocrine regulation of metamorphosis, and comparative life-history analysis, the study argues that both species exemplify non-teleological evolutionary pathways. By foregrounding strategy over deficiency, the article contributes to broader debates on normativity, progress, and directionality in evolutionary biology.

## Introduction

The persistence of larval characteristics into sexual maturity—a phenomenon I shall refer to here as neoteny, understood as one heterochronic pathway amongst several that can produce a paedomorphic phenotype—has long occupied an ambiguous position within evolutionary discourse. Whilst neoteny sensu stricto denotes the retention of juvenile somatic development alongside accelerated reproductive maturation, and whilst alternative routes such as progenesis can yield superficially similar outcomes, the cases examined here share a common pattern: developmental arrest coupled with aquatic habitat retention. For the purposes of this analysis, I employ “neoteny” as a functional descriptor of this syndrome, acknowledging that the underlying heterochronic mechanisms warrant careful empirical distinction in comparative contexts.

The phenomenon represents a striking departure from the canonical amphibian life cycle, wherein aquatic larvae undergo metamorphosis to become terrestrial or semi-terrestrial adults. Yet it invites interpretative frameworks that risk imposing normative hierarchies onto what are, fundamentally, value-neutral biological processes. The axolotl (*Ambystoma mexicanum*) and the olm (*Proteus anguinus*) serve as exemplary cases (Anan’eva Nb et al. 1988): both retain larval morphology throughout life, both inhabit highly specialised environments, and both challenge conventional assumptions about evolutionary trajectories.

The language surrounding neoteny has historically carried a teleological undertow, and traces of this framing can still be found in both specialist and popular accounts of amphibian development (see, e.g., Reilly et al. (1997)). Terms such as “arrested development,” “incomplete metamorphosis,” and “regressive evolution” carry implicit connotations of failure or suboptimality, as though these organisms represent evolutionary cul-de-sacs or developmental mistakes frozen in time. Such characterisations presuppose that metamorphosis constitutes the *telos* of amphibian ontogeny—a standard against which deviation must be measured and, invariably, found wanting. In those cases where this framing persists, it is not merely semantic; it risks structuring empirical investigation itself. When metamorphosis is implicitly assumed normative, research questions can become oriented towards identifying deficiencies rather than towards investigating alternative viable solutions on their own terms. When metamorphosis is assumed normative, research questions become oriented towards identifying deficiencies: what is “missing,” what has “failed to activate,” what prevents the organism from achieving its “proper” form. The epistemic consequence is that neotenic species are approached as problems requiring explanation rather than as viable solutions warranting investigation on their own terms.

The term ‘developmental arrest’ itself often betrays an ‘adultocentric’ bias, which erroneously presupposes that the terrestrial adult form is the teleological end-point of a linear process (Minelli 2011). As Minelli argues, animal development should be viewed as an open-ended segment of life where every stage possesses its own organisational integrity. Following Silvestros (Silvestros 2023) and Bich and Skillings (Bich and Skillings 2023), there are no ‘intermediate’ stages in a biological sense; the larval axolotl or olm is not an ‘incomplete’ organism, but a fully integrated and functional biological system whose ‘arrest’ is only a perceived state relative to a subjective morphological norm.

This article seeks to invert that interpretative lens. Rather than treating neoteny as a deficiency requiring explanation or apology, I propose that developmental arrest in the axolotl and the olm be reconsidered as a coherent evolutionary strategy—one that emerges from, and is exquisitely suited to, the ecological contexts these species have historically inhabited. Such a reframing necessitates engagement with several intersecting domains: the endocrine mechanisms governing metamorphosis, the evo-devo literature on developmental plasticity and constraint, life-history theory, and the broader philosophical questions concerning normativity in evolutionary explanation.

The axolotl, endemic to the remnant lake systems of the Valley of Mexico, and the olm, restricted to the subterranean karst waters of the Dinaric Alps, share more than their neotenic phenotype. Both occupy environments characterised by comparatively stable aquatic conditions, relatively reduced predation pressure compared to many surface habitats (though this has shifted dramatically for the axolotl following twentieth-century introductions of invasive fish species), and functional constraints on terrestrial existence—absolute in the case of the perpetually dark, humid cave systems inhabited by the olm, more contextually variable for the historically fishless, vegetation-rich lakes of the axolotl’s native range. Both exhibit reduced sensory structures—most notably, vestigial or absent eyes in the olm—and both have attracted considerable attention as model organisms, albeit for different reasons. The axolotl has become a cornerstone of regenerative biology, whilst the olm stands as an icon of cave-adapted vertebrates. Yet their shared retention of larval traits invites comparative analysis that transcends the boundaries of their respective research traditions.

Central to this investigation is the recognition that metamorphosis is not an evolutionary imperative but a conditional life-history transition, mediated by thyroid hormone signalling and subject to modulation by ecological variables. The capacity to undergo metamorphosis remains latent in many neotenic species, including the axolotl, which can be induced to metamorphose through exogenous thyroid hormone administration. This plasticity suggests that neoteny is not the result of genetic incapacity but rather reflects evolutionary modification of endocrine sensitivity, threshold values, and tissue responsiveness to hormonal signals. Selection operates not on the binary presence or absence of metamorphic capacity, but on the regulatory architecture governing when, whether, and how completely developmental transitions occur. In environmentally stable aquatic niches, selection may favour reduced thyroid hormone production, diminished receptor sensitivity, or altered downstream signalling cascades—effectively recalibrating the system such that metamorphosis becomes conditional, facultative, or functionally absent. This represents not developmental failure but developmental evolution: the fine-tuning of ontogenetic responsiveness to ecological circumstance.

In what follows, I shall first examine the endocrine and developmental mechanisms underpinning neoteny, situating them within the broader context of heterochrony and developmental evolution. I shall then turn to the specific ecologies of the axolotl and the olm, exploring how environmental parameters have rendered larval persistence not merely viable but advantageous under the conditions in which these lineages diversified. Subsequently, I shall address the concept of “regressive evolution,” arguing not for its abandonment—the term retains descriptive utility in denoting trait loss or simplification—but for its conceptual detoxification: the elimination of tacit assumptions that structural reduction entails adaptive inferiority. Finally, I shall consider the implications of this reinterpretation for our understanding of evolutionary strategy, adaptability, and the contested notion of progress in evolution. Throughout, the aim is not to reject metamorphosis as an adaptive solution but to resist its elevation as the sole or superior trajectory—to insist, in other words, that evolution admits of multiple solutions to the problem of existence, none of which can be judged deficient except against arbitrarily imposed standards.

## Heterochrony, Endocrine Regulation, and the Developmental Basis of Neoteny

The developmental mechanisms underlying neoteny are best understood within the broader framework of heterochrony—alterations in the timing or rate of developmental processes relative to ancestral ontogenetic patterns. Gould’s seminal work on heterochrony (Gould 1977) established a taxonomic vocabulary for such changes, distinguishing between processes that affect somatic development and those that influence reproductive maturation. Neoteny, in this schema, represents the retardation of somatic development whilst reproductive development proceeds at an ancestral or accelerated rate, yielding sexually mature individuals that retain juvenile morphological characteristics. This stands in contrast to progenesis, wherein reproductive maturation is accelerated whilst somatic development proceeds at a relatively normal rate, and to other heterochronic modes such as hypermorphosis or post-displacement.

It bears emphasising that Gould’s framework classifies developmental outcomes rather than identifying causal mechanisms. The typology describes what has changed in the timing of developmental events, not how those changes are achieved at the level of gene regulation, hormonal signalling, or cellular differentiation. This distinction is crucial: to understand neoteny as an evolutionary phenomenon, we must move beyond phenotypic description to examine the proximate developmental systems whose modification generates heterochronic patterns.

While Gould’s (Gould 1977) seminal work remains the primary touchstone for contemporary discussions, it is essential to acknowledge the foundational contributions of Gavin de Beer. In *Embryology and Evolution* (de Beer 1930) and later *Embryos and Ancestors* (de Beer 1958), de Beer provided the critical framework for understanding how developmental timing shifts could drive macroevolutionary change, directly inspiring the subsequent synthesis of ontogeny and phylogeny.

In amphibians, metamorphosis is orchestrated by a precisely regulated endocrine cascade dominated by thyroid hormones—principally thyroxine (T$_4$) and its more active derivative, triiodothyronine (T$_3$). The initiation and progression of metamorphosis depend upon rising titres of these hormones, which bind to thyroid hormone receptors (TRs) in target tissues, triggering transcriptional programmes (Shi 2000; Tata 2006) that govern tissue remodelling, apoptosis of larval structures, and differentiation of adult features. Tissue-specific responses are further modulated by local deiodinase activity, which regulates the peripheral conversion of T$_4$ to the more potent T$_3$, allowing fine-grained control (Tata 2006) over the timing and intensity of metamorphic changes in different anatomical regions. The system exhibits remarkable tissue-specificity: the same hormonal signal induces tail resorption in tadpoles whilst simultaneously promoting limb development, intestinal shortening, and restructuring of the nervous system.

In neotenic species, this endocrine system has been evolutionarily modified such that metamorphosis either fails to occur spontaneously or occurs only under exceptional circumstances. The mechanistic basis of this modification varies across taxa and has been the subject of extensive investigation, particularly in the axolotl. Early experimental work by Laufberger (Laufberger 1913), later discussed and contextualised in Soviet herpetological and endocrinological literature (Darevskij and Orlov 1988; Anan’eva Nb et al. 1988), demonstrated that axolotls could be induced to metamorphose through administration of thyroid hormones. This finding was pivotal: it demonstrated that neoteny in the axolotl does not result from the wholesale loss of metamorphic machinery but from alterations in its regulation.

Expanding beyond a strictly Neo-Darwinian focus on the survival of the fittest, an evo-devo perspective emphasizes the ‘arrival of the fittest’—that is, the inherent evolvability of the neotenic phenotype. The frequent occurrence of facultative neoteny (Whiteman 1994) across various caudate populations suggests that this developmental shift is not a drastic macroevolutionary leap, but a highly accessible transition (Whiteman 1994). By modulating the sensitivity of the hypothalamic-pituitary-thyroid (HPT) axis, these organisms can bypass metamorphosis through relatively minor developmental modifications. This demonstrates that neoteny is a robust and readily available evolutionary trajectory, facilitated by the existing plasticity of the amphibian developmental programme.

Subsequent research has identified multiple nodes at which this regulatory system can be modified. In the axolotl, reduced production of thyroid-stimulating hormone (TSH) from the pituitary appears to be a primary factor, resulting in chronically low circulating levels of thyroid hormones (Denver 2009; Shi 2000). Additionally, there is evidence for altered tissue sensitivity to thyroid hormones in some neotenic populations, suggesting evolutionary modification of receptor density, receptor isoform expression, or downstream signalling components. The Mexican axolotl exhibits particularly low thyroid hormone levels throughout ontogeny, and whilst induced metamorphosis is possible, it frequently results in individuals with compromised viability—a finding that underscores the integrated nature of developmental systems and the difficulty of disentangling elements that have co-evolved over millions of years.

The olm presents a somewhat different picture. Unlike the axolotl, attempts to induce metamorphosis in *Proteus anguinus* through thyroid hormone administration have yielded equivocal or negative results (Shi 2000), suggesting more profound evolutionary modifications to the metamorphic programme. This pattern is consistent with the antiquity of cave adaptation in this lineage—estimated at several million years—and may reflect the concomitant relaxation of selection pressures that might otherwise maintain metamorphic capacity. It is, however, pertinent to distinguish between the facultative neoteny observed in *A. mexicanum* and the strictly obligate condition of *P. anguinus*. Whilst the former retains the latent physiological capacity to metamorphose under exogenous hormonal stimulation, the latter appears to have undergone a definitive ontogenetic decoupling, representing a more absolute evolutionary commitment to the aquatic larval form. In environments where terrestrial existence is functionally impossible and where the larval phenotype is demonstrably viable across the entire lifespan, mutations that compromise metamorphic competence incur no fitness penalty and may even prove advantageous by eliminating the metabolic costs associated with maintaining unused developmental machinery. Furthermore, this ‘regressive’ trajectory is often underpinned by a stringent cost-benefit logic related to metabolic efficiency. In the resource-scarce subterranean ecosystems of the olm, the systematic reduction of energetically expensive tissues—such as ocular structures and complex pigmentation—functions as a crucial adaptive optimisation of limited energy reserves. The empirical evidence for such cost savings remains somewhat indirect, but the logic is evolutionarily robust.

It is worth emphasising that the distinction between regulatory modification and structural loss is not absolute but represents a continuum. Early in the evolution of neoteny, lineages likely retain full metamorphic competence, with heterochrony achieved purely through endocrine modulation—alterations in hormone production, timing, or tissue responsiveness that leave the downstream machinery intact. Over evolutionary time, however, relaxed selection on metamorphic structures permits the accumulation of mutations that would be deleterious in metamorphosing lineages. Genes encoding matrix metalloproteinases involved in tail resorption, transcription factors governing eyelid morphogenesis, or keratin isoforms specific to terrestrial epidermis may acquire loss-of-function mutations, become pseudogenised, or experience regulatory decay. The system moves from conditional to obligate neoteny, from facultative to constitutive retention of larval traits.

This temporal dimension is crucial for interpreting patterns of developmental evolution. The axolotl, whose neoteny may be relatively recent in evolutionary terms and whose metamorphic capacity remains inducible, occupies one position on this continuum. The olm, with its deeper evolutionary roots in subterranean environments and its apparent loss of metamorphic competence, occupies another. Yet both represent evolutionarily stable solutions to the challenge of persisting in aquatic habitats, differing in degree rather than kind.

From an evo-devo perspective, neoteny exemplifies the principle that evolution operates through modification of developmental timing and regulation rather than through invention of novel structures de novo. The larval amphibian body plan—with external gills, lateral line systems, caudal fins, and aquatic sensory apparatus—represents a fully functional, ecologically viable morphology. Metamorphosis evolved as an adaptation enabling exploitation of terrestrial or semi-terrestrial niches, but it is not a prerequisite for survival per se.

In lineages where ecological circumstances favour or permit continuous aquatic existence, selection may act to suppress, delay, or eliminate metamorphosis, effectively “locking in” the larval phenotype as the adult form. This has profound implications for how we conceptualise developmental pathways. Rather than viewing ontogeny as a unidirectional trajectory from embryo through larva to obligate adult, we might better conceive of it as a branching developmental landscape wherein multiple endpoints are possible, each contingent upon environmental signals, endocrine states, and selective regimes. Neoteny does not represent departure from a developmental “programme” so much as selection of an alternative route through developmental space—a route that, under appropriate ecological conditions, may be as adaptive as any other.

The endocrine plasticity observed in amphibians more broadly supports this interpretation. Many salamander species exhibit facultative neoteny (Whiteman 1994; Bonett and Chippindale 2006), wherein some individuals undergo metamorphosis whilst others in the same population retain larval characteristics, often in response to environmental variables such as pond permanence, food availability, or population density. This intraspecific variation demonstrates that the developmental decision to metamorphose or remain neotenic can be ecologically labile, governed by threshold responses to environmental cues mediated through the thyroid axis. Obligate neoteny, as seen in the axolotl and olm, represents the evolutionary fixation of one outcome within this conditional system—a canalisation of development along a pathway that was ancestrally plastic.

Understanding neoteny as a consequence of heterochrony enacted through endocrine modification thus provides a mechanistic foundation for reinterpreting its adaptive significance. It is not that development has “stopped” or “failed,” but rather that it has been evolutionarily redirected, with selection acting on the regulatory parameters governing when and whether particular developmental transitions occur. The retention of larval characteristics is not arrested development but alternative development—a distinction with considerable theoretical weight.

## Ecological Context and Adaptive Landscapes: The Niches of the Axolotl and the Olm

To argue that neoteny represents an adaptive evolutionary strategy—understood here as a population-level outcome of selection rather than an organism-level “choice”—requires demonstration that the environments inhabited by neotenic species render larval persistence advantageous, or at minimum non-disadvantageous, relative to metamorphosis. The axolotl and the olm occupy markedly different ecological settings, yet both exhibit environmental characteristics that favour continuous aquatic existence and potentially select against the costs associated with metamorphic transformation. This section examines the specific ecological contexts of these two species, exploring how habitat structure, resource availability, predation regimes, and physical constraints shape the adaptive value of developmental arrest.

### The Axolotl: Persistence in an Ephemeral Lacustrine System

*Ambystoma mexicanum* is endemic to a highly circumscribed geographic range: historically, the interconnected lake system of the Valley of Mexico, including Lake Xochimilco and Lake Chalco. This distribution is notable for its extreme restriction—the axolotl represents one of the most geographically limited vertebrate species—and for the specific limnological characteristics of its native waters. Prior to extensive anthropogenic modification, these lakes were characterised by relatively stable water levels, abundant aquatic vegetation, and a fishless or fish-poor vertebrate fauna (Zambrano et al. 2010; Voss et al. 2009). The absence of large predatory fish is particularly significant, as fish predation represents one of the primary selective pressures favouring metamorphosis in many salamander lineages.

The lacustrine habitat of the axolotl provides year-round aquatic conditions with sufficient depth, oxygen availability, and structural complexity to support a fully aquatic lifecycle. Neotenic individuals retain larval characteristics optimised for this environment: external gills with high surface area for efficient oxygen extraction, a laterally compressed tail facilitating aquatic locomotion, and a functional lateral line system for prey detection and spatial orientation in turbid water. These features confer clear adaptive advantages in the axolotl’s native habitat, where foraging occurs primarily in soft sediments and amongst submerged vegetation.

Metamorphosed *Ambystoma* species, by contrast, exhibit morphological adaptations for terrestrial or semi-terrestrial existence: loss of external gills and lateral line organs, development of eyelids and corneal modifications for aerial vision, alterations in skin structure including increased keratinisation and mucus gland density, and skeletal remodelling that affects locomotor mechanics. In an environment where terrestrial habitat is limited to narrow, often inundated margins and where aquatic resources are abundant and predictably available, these metamorphic transformations offer minimal adaptive benefit whilst incurring substantial energetic costs.

The energetics of metamorphosis are non-trivial. The process involves widespread tissue apoptosis, cellular proliferation, and extensive morphological reorganisation—all metabolically expensive. From a life-history perspective, this represents a fundamental allocation trade-off: energy diverted toward metamorphic reorganisation is energy unavailable for somatic growth, immune function, or reproductive investment. For a population inhabiting a stable aquatic environment with no seasonal desiccation pressure and no requirement for terrestrial dispersal, the fitness returns on this energetic investment are unclear. Moreover, metamorphosed individuals must cope with the physiological demands of terrestrial gas exchange, water balance, and thermoregulation—challenges that neotenic individuals, remaining aquatic, largely circumvent.

It must be acknowledged that the contemporary ecological context of the axolotl differs dramatically from historical conditions. The introduction of invasive fish species, particularly carp (*Cyprinus carpio*) and tilapia (*Oreochromis* spp.), has fundamentally altered predation dynamics, contributing to the near-extinction of wild axolotl populations (Zambrano et al. 2010; Voss et al. 2009). Urban expansion has led to severe habitat degradation, pollution, and hydrological modification. Under these novel and deteriorating conditions, neoteny may no longer confer the adaptive advantages it did historically. However, the evolution of obligate neoteny in the axolotl lineage occurred over timescales preceding recent anthropogenic impacts, under ecological conditions that would have favoured continuous aquatic existence. Evolutionary explanations must be historically situated, assessed against the selective regimes that shaped trait evolution rather than against contemporary conditions that postdate the relevant evolutionary transitions.

### The Olm: Adaptation to Perpetual Subterranean Darkness

*Proteus anguinus* inhabits a radically different environment: the karstic cave systems and subterranean aquifers of the Dinaric Alps, extending through Slovenia, Croatia, Bosnia and Herzegovina, and northeastern Italy. This habitat represents one of the most extreme environments occupied by any vertebrate: perpetually dark, thermally stable, nutrient-poor, and entirely devoid of opportunities for terrestrial existence. Cave ecosystems impose unique selective pressures, and the olm exhibits a suite of adaptations reflecting prolonged evolutionary residence in this habitat.

The most conspicuous of these is the vestigial or entirely absent eyes, which lie beneath translucent skin and lack functional visual capability. This trait exemplifies what is conventionally termed regressive evolution—the reduction or loss of structures that impose maintenance costs without conferring fitness benefits in environments where their function is obsolete. I employ the term “regressive” here in a strictly functional sense, denoting directional change toward structural simplification, without normative implication regarding adaptive value. In perpetual darkness, investment in visual system development represents pure metabolic expense with no compensating advantage. Selection thus favours mutations that reduce or eliminate such investment, channelling resources toward sensory modalities relevant to subterranean life.

The olm has evolved enhanced mechanoreceptive and chemoreceptive capabilities, mediated by an elaborate lateral line system and ampullary electroreceptors (Schlegel and Bulog 1997; Istenič and Bulog 1984)—the latter a rare trait among amphibians, convergently evolved with certain fish lineages inhabiting similar environments (Wilkens 2010). These sensory adaptations are features of the larval amphibian body plan, retained and potentially elaborated in the neotenic phenotype. Metamorphosis, which would entail loss of the lateral line system, would thus eliminate precisely those sensory structures most valuable in the olm’s ecological context—a clear case in which developmental “progression” toward the metamorphic phenotype would constitute functional regression in adaptive terms.

The subterranean aquatic environment also imposes severe energetic constraints. Cave ecosystems are typically oligotrophic, with primary productivity limited to chemosynthetic bacteria and organic material imported from surface systems via percolating groundwater (Culver and Pipan 2009). Food availability is unpredictable and often scarce. Under such conditions, the metabolic costs of metamorphosis would be particularly onerous, potentially exceeding the energetic reserves available to individuals. The olm exhibits an extraordinarily slow life history, with estimates of sexual maturity not achieved until 15–20 years of age and potential lifespans exceeding a century (Voituron et al. 2011; Gorički et al. 2012). This extreme longevity and delayed reproduction are characteristic of organisms inhabiting resource-limited environments (Culver and Pipan 2009), where the primary challenge is not rapid growth and reproduction but long-term survival through periods of scarcity.

Moreover, the physical structure of the olm’s habitat renders terrestrial existence functionally impossible. Cave systems offer no terrestrial refugia, no seasonally available land surfaces, and no ecological niches that would reward the morphological transformations associated with metamorphosis. The aquatic habitat is permanent, stable, and inescapable. Under these conditions, any genetic architecture predisposing individuals toward metamorphosis would be selectively neutral at best and potentially maladaptive if metamorphic development proceeded without functional endpoint.

### Convergent Selection on Neoteny in Disparate Environments

Despite the profound ecological differences between shallow, vegetation-rich lakes and deep, oligotrophic cave systems, both environments share key features that favour neoteny. Both provide permanent aquatic habitat, obviating the need for amphibious or terrestrial life stages. Both historically featured reduced predation pressure from large aquatic predators, eliminating one of the primary selective advantages of metamorphosis: escape from predator-rich larval habitats to safer terrestrial refugia. Both reward retention of larval sensory and locomotor structures adapted for aquatic foraging and spatial navigation.

This convergence suggests that neoteny is not an idiosyncratic or aberrant outcome but a predictable evolutionary response to a particular constellation of ecological variables. Where aquatic habitats are stable, productive or at least sufficient, and unaccompanied by strong selective pressures favouring terrestrial capabilities, developmental pathways that circumvent or suppress metamorphosis may be actively favoured. The evolution of diverse life-history strategies in salamanders, including the loss of metamorphosis, is well-documented as a derived rather than a primitive condition (Bonett et al. 2013). The axolotl and the olm represent independent evolutionary experiments yielding similar solutions to analogous ecological problems.

It is instructive to contrast these cases with closely related species that do undergo metamorphosis. Other members of the genus *Ambystoma* exhibit facultative or obligate metamorphosis, often in habitats subject to seasonal drying or high aquatic predation. Similarly, surface-dwelling relatives of cave salamanders typically retain metamorphic competence, reflecting the continued adaptive value of terrestrial life stages in their respective environments. The evolutionary lability of metamorphosis within these clades—its presence in some lineages, suppression in others—underscores the contingent, context-dependent nature of its adaptive value. This pattern argues against any model of amphibian development that privileges metamorphosis as a universal endpoint or that treats neoteny as deviation from a normative developmental trajectory.

From a life-history perspective, neoteny can be understood as a strategy that is associated with fitness under specific ecological conditions under particular demographic and ecological constraints. In stable, aquatic environments with low extrinsic mortality, selection may favour allocation of resources toward somatic maintenance, enhanced foraging capability, and reproductive investment rather than toward the morphological reorganisation required for habitat transition. The retention of larval morphology is not developmental stasis but strategic allocation—a life-history decision encoded in developmental regulatory networks and shaped by ecological selection pressures.

This interpretation reframes neoteny from a passive failure to metamorphose into a coherent evolutionary outcome: the elaboration and retention of a phenotype well-suited to a particular ecological niche through selection. The axolotl and olm do not represent organisms “trapped” in larval form but rather organisms whose developmental programmes have been evolutionarily refined to produce adults optimally adapted to continuous aquatic existence. The larval phenotype is the adaptive adult phenotype in these contexts—a realisation that fundamentally challenges the normative privileging of metamorphosis as the amphibian telos.

## Regressive Evolution Reconsidered: Beyond Normative Frameworks

The concept of regressive evolution has long occupied a problematic position within evolutionary discourse. Ostensibly descriptive—denoting evolutionary change characterised by structural simplification, organ loss, or reduced complexity—the term carries inescapable normative overtones. “Regression” implies retreat from some anterior state of advancement, a backward movement along a presumed axis of progress. When applied to neoteny, eye loss in cave organisms, or the reduction of skeletal elements in parasitic lineages, the language of regression subtly frames these evolutionary outcomes as diminishments, as losses of something valuable or normative.

The framing of trait loss as ‘regressive’ is problematic as it implies a reversal of evolutionary progress. As McShea (McShea 1991) posits, while complexity may increase as a default state, natural selection frequently drives a reduction in complexity to enhance fitness within stable niches. In this context, the neotenic state represents what Lyons and Arbuckle (Lyons and Arbuckle 2024) term a ‘goldilocks zone’—an optimal fitness peak where the physiological costs of metamorphosis outweigh its benefits. Consequently, these transitions are more accurately characterised as ‘secondary simplifications’ rather than ‘regressions,’ thereby stripping the evolutionary narrative of its implicit, and often misleading, hierarchical value judgements.

McShea’s (1991) argument that complexity increases as a default state, with natural selection actively required to drive simplification, is fully consistent with this view: the neotenic transitions examined here are precisely instances where selection has acted, rather than cases of drift or default change. The proposed definition of ‘regressive evolution’ thus denotes the direction of change, not its mechanism or value.

This section argues for a conceptual detoxification of regressive evolution: the retention of the term’s descriptive utility whilst excising its normative baggage. Structural simplification is an evolutionary trajectory like any other, subject to the same processes of mutation, selection, and drift, and assessable only with reference to specific ecological contexts and fitness consequences. The olm’s vestigial eyes and the axolotl’s retention of larval structures are not evolutionary failures but adaptations—or at minimum, non-maladaptive outcomes—shaped by selective regimes that render complexity superfluous or costly.

### The Linguistic and Conceptual Heritage of “Regression”

The notion of regressive evolution is deeply entangled with nineteenth-century conceptions of evolutionary progress. Early evolutionary theorists, influenced by Lamarckian ideas of inherent developmental tendencies and Victorian notions of civilisational advancement, frequently interpreted evolution as a directional process moving toward increasing complexity, differentiation, and perfection. Organisms were situated along *scala naturae*-inflected hierarchies, with “higher” forms distinguished by morphological elaboration, physiological sophistication, and behavioural complexity.

Against this backdrop, any evolutionary trajectory characterised by simplification appeared anomalous, even pathological. Parasitic organisms that had lost locomotor structures, sensory organs, or digestive systems were described as “degenerate”. Cave-dwelling species with reduced eyes were characterised as exhibiting “retrogressive” evolution. The implicit framework was one of loss and decline, with complexity serving as both the telos and the measure of evolutionary success.

This conceptual architecture persists, albeit in attenuated form, within contemporary evolutionary biology. Whilst few modern biologists would explicitly endorse a linear model of evolutionary progress, the language of regression continues to imply deviation from an unstated norm. Terms such as “vestigial”, “rudimentary”, and “degenerate” carry connotations of incompleteness or obsolescence. Even ostensibly neutral descriptions—“reduced”, “simplified”, “lost”—acquire normative valence through their implicit contrast with “complete”, “complex”, or “present” structures presumed to represent the standard condition.

### Structural Simplification as Adaptive Outcome

A more theoretically defensible approach treats structural simplification not as regression but as adaptive modification in response to altered selective pressures. The evolutionary loss or reduction of traits occurs when the costs of maintaining those traits—metabolic, developmental, or otherwise—exceed their fitness benefits. This is not retreat but optimisation: the reallocation of limited resources away from functionless structures toward traits that enhance survival or reproduction in the organism’s actual ecological context.

Consider the olm’s eye reduction. In the perpetual darkness of subterranean aquifers, functional vision confers no advantage. Eyes impose developmental costs (the genetic machinery for eye formation, the cellular and molecular resources required for photoreceptor production), maintenance costs (metabolic expense of sustaining neural tissue and photopigments), and potentially opportunity costs (developmental programmes diverted toward vision cannot simultaneously be allocated elsewhere). Mutations that disrupt eye development, which would be strongly deleterious in surface-dwelling populations, are selectively neutral or even advantageous in cave populations. Over evolutionary time, such mutations accumulate, leading to progressive reduction and eventual loss of the visual system.

This is not degeneration but adaptation. The olm has not failed to develop eyes; it has evolved a developmental programme optimised for an environment where eyes are metabolically expensive irrelevancies. The resulting phenotype—eyeless, depigmented, with elaborated non-visual sensory systems—is precisely suited to its habitat. To describe this as regressive is to impose an arbitrary standard derived from surface-dwelling relatives and to privilege complexity for its own sake rather than evaluating traits in terms of their contribution to fitness.

A similar logic applies to neoteny. The retention of larval characteristics and the bypass of metamorphosis represent a specialised developmental trajectory. Traits such as external gills, cartilaginous skeletons, and lateral line systems are often framed as “simplified” relative to the terrestrial adult form, yet they constitute a highly effective morphological suite for permanent aquatic living. External gills, for example, facilitate efficient gas exchange, serving as a functional alternative to the lungs of terrestrial relatives. Similarly, reduced ossification aids in achieving neutral buoyancy, and the lateral line system provides sensory capabilities essential for underwater navigation. Consequently, these features are best understood as niche-optimised adaptations rather than markers of developmental deficiency or evolutionary regression.

Neoteny thus exemplifies a broader principle: evolutionary trajectories are shaped by functional demands, not by abstract hierarchies of complexity. Where environmental conditions favour simplicity, selection produces simplicity. Where they favour elaboration, selection produces elaboration. Neither trajectory is inherently progressive or regressive; both are contingent responses to ecological circumstance.

### Dollo’s Law and the Irreversibility of Evolution

A related conceptual challenge concerns the perceived irreversibility of evolutionary change, encapsulated in Dollo’s Law: the principle that complex structures, once lost, are unlikely to re-evolve in identical form. This principle is often invoked to characterise regressive evolution as a one-way street, a descent into simplification from which lineages cannot return. The implication is that regression represents an evolutionary dead end, foreclosing future adaptive possibilities.

Dollo’s Law is, however, a probabilistic generalisation rather than a mechanistic constraint. It reflects the astronomical improbability of re-evolving identical genetic architectures, developmental programmes, and phenotypic structures through independent mutational pathways. It does not imply that lineages undergoing structural simplification are evolutionarily impoverished or that they lack adaptive potential. Cave fishes that have lost eyes can still evolve novel sensory adaptations, altered foraging behaviours, and modified life histories. Neotenic salamanders retain the developmental plasticity to respond to new selective pressures, even if that response does not involve re-evolution of the specific metamorphic programme lost in their ancestors.

Moreover, Dollo’s Law applies equally to all evolutionary change, not merely to simplification. A lineage that evolves a complex novel structure—say, the vertebrate eye or the insect wing—is equally unlikely to evolve that structure a second time in identical form should it be lost. The irreversibility is a consequence of the contingency and path-dependence of evolution, not a special feature of regressive trajectories. To invoke Dollo’s Law selectively as evidence of the problematic nature of regression is to misunderstand its scope and implications.

### Complexity, Constraint, and Evolvability

A subtler argument holds that structural simplification reduces evolvability—the capacity of a lineage to generate heritable phenotypic variation upon which selection can act. By this reasoning, organisms that have lost complex structures have fewer developmental “modules” available for evolutionary tinkering, constraining their ability to adapt to future environmental changes. Neotenic salamanders, having suppressed metamorphosis, might be less able to colonise terrestrial habitats should aquatic environments become unavailable.

This argument has superficial plausibility but conflates two distinct concepts: current adaptive value and future adaptive potential. Evolution does not optimise for hypothetical future contingencies; it responds to immediate selective pressures. An organism living in a stable aquatic environment experiences no selection favouring retention of metamorphic competence “just in case” terrestrial habitats become relevant in the future. Selection operates on present fitness differentials, not on speculative scenarios.

Furthermore, the premise that complexity enhances evolvability is itself contestable. Highly integrated, complex systems may be developmentally constrained, with mutations in one component producing deleterious pleiotropic effects elsewhere. Simpler systems, with fewer functional interdependencies, may permit greater developmental flexibility. The axolotl’s regenerative capacity—among the most remarkable within vertebrates (Tanaka and Reddien 2011)—is facilitated by aspects of its neotenic phenotype, although it is surpassed by the structural regeneration seen in invertebrates such as hydra or planarians.

Evolvability is not a monotonic function of structural complexity. It depends on genetic architecture, developmental modularity, mutation rates, population size, and the specific selective challenges a lineage faces. To assume that simplification inherently reduces evolvability is to make an unwarranted generalisation unsupported by comparative evidence.

### Towards a Value-Neutral Conception of Evolutionary Trajectories

If regressive evolution is to retain analytical utility, it must be redefined in strictly operational terms, stripped of normative connotations. I propose the following: regressive evolution denotes evolutionary change characterised by reduction in the number, size, or complexity of morphological structures, assessed relative to an ancestral state reconstructed through phylogenetic analysis. This definition is descriptive and comparative. It specifies a direction of change without implying that the change is maladaptive, suboptimal, or evolutionarily problematic.

Under this definition, the olm’s eye loss is regressive—eyes present in ancestors are reduced or absent in descendants. The axolotl’s neoteny involves regressive changes—loss of metamorphic structures present in ancestral *Ambystoma* lineages (Bonett et al. 2013; Lyons and Arbuckle 2024). But these are simply descriptions of evolutionary trajectories, no different in kind from descriptions of trait elaboration or stasis. The evaluative work—determining whether these changes are adaptive, neutral, or maladaptive—must be done separately, through ecological and functional analysis of the sort undertaken in Section 3.

This reframing has significant implications for how we interpret evolutionary patterns. Neoteny is not developmental arrest or failure to complete a normative ontogenetic programme; it is an alternative developmental outcome with its own adaptive logic. Eye loss in cave organisms is not degeneration but resource reallocation. Structural simplification is not evolutionary decline but niche-specific optimisation. Progress and regression, as evaluative categories, dissolve under scrutiny. What remains are evolutionary trajectories, each contingent, each adaptive (or neutral, or maladaptive) only relative to specific environmental contexts, and none privileged by nature itself.

## Implications and Broader Contexts: Normativity, Diversity, and the Meaning of Adaptation

The foregoing analysis of neoteny in the axolotl and olm carries implications extending beyond amphibian developmental biology. By demonstrating that structural simplification and developmental arrest can constitute viable life-history configurations rather than deficiencies, this study participates in a broader intellectual project: the critique of normative frameworks that privilege certain phenotypes, developmental trajectories, or evolutionary outcomes as standard whilst pathologising deviations as failures, losses, or regressions. This final section situates the biological arguments within wider theoretical contexts, exploring resonances with debates in disability studies, neurodiversity frameworks, and the cultural politics of difference.

### Biology and Normativity: The Problem of the “Typical”

Biological discourse is pervaded by implicit normative standards. We speak of “normal” development, “typical” morphology, and “wild-type” genotypes, establishing reference points against which variation is measured. These standards are ostensibly descriptive—normal as statistical mode, typical as most frequent—but they function prescriptively, rendering deviations intelligible primarily as departures from an unmarked norm. The language of deficit follows naturally: atypical becomes abnormal, variant becomes deficient, different becomes defective.

This normalising impulse is not confined to discussions of human biology but structures comparative and evolutionary biology as well. The metamorphosed amphibian serves as the implicit standard against which neotenic species are evaluated; the sighted organism as the baseline rendering eyeless cave dwellers noteworthy. Yet these standards are arbitrary, products of phylogenetic happenstance and the historical accidents of which organisms became model systems for biological research. There is no biological warrant for privileging metamorphosis over neoteny, vision over blindness, complexity over simplicity—only the contingent fact that certain phenotypes are more familiar, more common in the organisms humans encounter, or more amenable to laboratory study.

The axolotl and olm serve as instructive counterexamples precisely because their “atypical” phenotypes are demonstrably adaptive within their ecological contexts. Their developmental trajectories are not deviations from a proper programme but alternative programmes, shaped by different selective pressures and producing different—not inferior—functional outcomes. This realisation invites reconsideration of how we conceptualise biological variation more generally. If neoteny is not developmental failure, if eye loss is not degeneration, then the evaluative frameworks we apply to biological diversity require fundamental reassessment.

### Parallels with Disability Studies and Neurodiversity Frameworks

The conceptual move from deficit to difference finds strong parallels in disability studies and neurodiversity scholarship. These fields have articulated powerful critiques of medical and social models that pathologise human variation, arguing instead for frameworks that recognise diverse embodiments and neurotypes as valid modes of being rather than deviations from able-bodied or neurotypical norms.

The social model of disability, for instance, distinguishes between impairment (biological difference) and disability (social disadvantage arising from environments designed for a narrow range of bodies and minds). A person using a wheelchair is not inherently disabled by lower-limb paralysis but is disabled by architectural environments constructed around assumptions of bipedal locomotion. The “problem” resides not in the body but in the mismatch between body and environment. Redesign the environment—provide ramps, lifts, accessible transport—and disability diminishes or disappears.

This framework resonates strongly with the ecological analysis of neoteny. The axolotl is not disabled by its retention of gills and larval morphology; it is exquisitely adapted to aquatic environments. It becomes “deficient” only when assessed against environments—terrestrial habitats—for which it was not selected and in which it does not naturally occur. The perceived deficit is a product of applying inappropriate evaluative standards, of measuring the organism against ecological contexts irrelevant to its evolutionary history.

Similarly, neurodiversity advocates argue that conditions such as autism are not disorders requiring cure but neurological variations with distinct cognitive profiles, strengths, and adaptive challenges (Armstrong 2010; Chapman 2019). As I have argued elsewhere from an autiethnographic perspective, autism is best understood as a natural variant within the human species—a product of nature’s ceaseless diversification rather than a developmental deviation from a fixed norm (Mussies 2024). The “deficits” associated with autism—difficulties with social communication, sensory sensitivities, preference for routine—are deficits only relative to neurotypical social environments (Milton 2012). In contexts valuing focused attention, pattern recognition, and systematic thinking, autistic cognitive styles may be advantageous (Armstrong 2010). The evaluative judgement depends entirely on what is valued and what environmental demands are foregrounded.

The axolotl’s lateral line system, useless in terrestrial contexts, is invaluable for aquatic navigation. The olm’s blindness, catastrophic on the surface, is irrelevant in perpetual darkness and permits resource reallocation toward functional sensory modalities. Adaptive value is always context-dependent, and contexts are multiple. There is no universal standard of function, only local optima shaped by particular ecological or social niches.

### Evolutionary Biology and the Spectre of Progress

The reluctance to abandon normative hierarchies in biology reflects, in part, lingering attachments to notions of evolutionary progress. Despite widespread acknowledgment among biologists that evolution is non-teleological and that natural selection optimises only for local, immediate fitness rather than long-term advancement, intuitions about directionality persist. Complexity, intelligence, size, metabolic rate—various candidates have been proposed as axes along which evolution exhibits directional trends, with humans predictably situated near the apex.

These intuitions are difficult to dislodge because they align with culturally pervasive narratives of improvement and advancement. Progress is a foundational concept in Western modernity, structuring understandings of history, technology, and human development. To assert that evolution lacks direction, that bacteria are as “advanced” as mammals in the only sense that matters evolutionarily (both are well-adapted to their respective niches), feels counterintuitive, even threatening to anthropocentric self-conceptions.

Yet the evidence overwhelmingly supports a non-progressive view. Evolutionary success is measured solely by persistence—the ability of lineages to survive and reproduce over time. By this metric, bacteria are wildly successful, having persisted for billions of years across every conceivable environment. Parasitic organisms, often dismissed as degenerate, are astonishingly diverse and abundant, their structural simplifications enabling exploitation of niches unavailable to more “complex” free-living relatives. Cave organisms, including the olm, occupy extreme environments and exhibit specialised adaptations reflecting millions of years of evolutionary refinement.

The axolotl and olm do not represent evolutionary failures or dead ends. They represent evolutionary successes: lineages that have persisted, diversified (in the case of other neotenic salamanders), and continue to occupy their respective niches. Their neoteny is not arrested development but completed development along an alternative trajectory. To insist otherwise is to impose a teleological framework foreign to evolutionary processes themselves.

### Cultural Narratives and the Politics of “Loss”

Beyond academic biology, narratives of loss and regression carry cultural and political weight. Discourses of civilisational decline, genetic degeneration, and cultural decay have historically been mobilised to justify exclusionary politics, eugenic interventions, and hierarchies of human worth. The language of regressive evolution, even when applied to non-human organisms, participates in a broader symbolic economy wherein simplification is coded as decline, difference as deficit, and deviation as pathology.

These associations are neither inevitable nor natural. They are culturally constructed and historically contingent, reflecting particular value systems rather than biological realities. To describe the olm’s eyelessness as regression is to privilege sightedness as normative, an evaluative stance without biological foundation. To characterise neoteny as developmental arrest is to assume that metamorphosis represents completion, again an arbitrary standard.

Recognising these framings as normative rather than descriptive opens space for alternative interpretations. The olm’s sensory ecology can be understood as a sophisticated adaptation to subterranean life rather than as loss of vision. The axolotl’s neoteny can be appreciated as an elegant solution to the challenge of aquatic persistence rather than as failure to metamorphose. These reframings do not deny difference—the olm is blind, the axolotl does not metamorphose—but they refuse to code difference as deficiency.

This refusal has broader implications. If evolutionary biology can dispense with normative hierarchies, recognising multiple adaptive solutions as equally valid outcomes of evolutionary processes, it provides conceptual resources for challenging normative hierarchies in other domains. The diversity of developmental trajectories in amphibians becomes a model for thinking about human diversity: neurological, physical, cognitive, and otherwise. Not as deviations from a standard to be corrected or cured, but as variation to be accommodated, understood, and valued on its own terms.

### Towards Pluralistic Conceptions of Adaptation

The central argument of this article is that neoteny and regressive evolution, properly understood, represent evolutionary solutions rather than developmental deficiencies. Before proceeding, however, one methodological clarification deserves explicit acknowledgement. The preceding sections have compared species—the axolotl and the olm—representing an interspecific comparison. The move in this section to cases of human neurological and physical diversity involves an extension from interspecific to intraspecific comparison. This extension is legitimate—the same logic of context-dependence and adaptive pluralism applies—but it is not trivial. Variation among species and variation within a species operate through partly different mechanisms and are subject to different evolutionary constraints. The analytical parallels drawn here are conceptual rather than strictly biological, and should be understood as heuristic rather than homologous.

A useful intermediate case that bridges these scales is that of social insect colonies with morphological castes. In species such as the leafcutter ant *Atta cephalotes* or the army ant *Eciton burchellii*, the same genome generates dramatically different adult phenotypes—minor workers, major workers (soldiers), and reproductives—that coexist within a single colony (Wilson 1971; Hölldobler and Wilson 1990). The phenotype of a minor worker is not better or worse than that of a soldier; each is functionally specialised for distinct colony-level tasks, and the overall fitness of the colony depends on the maintenance of phenotypic diversity rather than the convergence of all individuals toward a single “optimal” form. No caste can be described as developmentally arrested relative to another; each represents a complete, integrated phenotype shaped by developmental plasticity in response to colony-internal signals. This intraspecific case illustrates the same principle that the interspecific axolotl–olm comparison demonstrates: that multiple phenotypic configurations can simultaneously represent viable, adaptive outcomes, and that evaluating any one configuration as deficient requires an arbitrary privileging of a single type.

This perspective entails adopting a radically pluralistic conception of adaptation. Rather than seeking to identify the “best” or most “advanced” solutions to evolutionary challenges, we should recognise that challenges themselves are multiple and that solutions are correspondingly diverse. Aquatic and terrestrial environments pose different selective pressures; metamorphosis is adaptive in the latter context, neoteny in the former. Light-filled and lightless environments reward different sensory investments; vision is adaptive where photons are available, alternative modalities where they are not.

Pluralism of this sort does not entail relativism. It is not the case that all phenotypes are equally adaptive in all contexts—that would be absurd. Rather, it is that adaptive value can only be assessed relative to specific ecological contexts and that those contexts are irreducibly various. There is no view from nowhere, no universal standard of fitness transcending particular environments and their attendant selection pressures.

This philosophical stance aligns with contemporary developments in evolutionary theory, including niche construction theory, extended evolutionary synthesis frameworks, and critiques of adaptationism. These approaches emphasise the contingency, context-dependence, and multiplicity of evolutionary outcomes, resisting reductive accounts that privilege single mechanisms, universal trends, or teleological endpoints. The case studies examined here—the axolotl and the olm—exemplify this complexity, demonstrating that even closely related phenomena (neoteny in two salamander lineages) arise through different mechanisms, respond to different ecological pressures, and produce different degrees of developmental modification.

### Conclusion: Rewriting the Narrative

Neoteny in the axolotl and the olm is not a curiosity, an anomaly, or an evolutionary dead end. It is a testament to the flexibility of developmental systems, the power of ecological context to shape evolutionary trajectories, and the absence of inherent directionality in evolution. By reinterpreting developmental arrest as adaptive outcome and structural simplification as functional optimisation, this article challenges normative frameworks that privilege complexity, completeness, and progression as evolutionary goods.

The implications extend beyond amphibian biology. They invite reconsideration of how we conceptualise diversity, difference, and adaptation across biological and social domains. They provide grounds for resisting narratives of deficit and decline, offering instead frameworks that recognise multiple valid solutions to the challenges of existence. And they remind us that the language we use to describe biological phenomena is never neutral, always carrying implicit values that shape both scientific inquiry and broader cultural understandings.

The axolotl, with its feathery gills and regenerative prowess, and the olm, ghostly and eyeless in its subterranean realm, are not failed metamorphs or degenerate cave dwellers. They are evolutionary achievements: organisms superbly adapted to their respective niches, embodying alternative developmental logics, and challenging our preconceptions about what counts as biological success. In learning to see them on their own terms, we may learn to see diversity itself differently—not as deviation from a norm, but as the very fabric of life’s adaptive richness.

## Data Availability

No datasets were generated or analysed during the current study.
